# Thinking by metaphor, fast and slow: Deliberate Metaphor Theory offers a new model for metaphor and its comprehension

**DOI:** 10.3389/fpsyg.2023.1242888

**Published:** 2023-09-05

**Authors:** Gerard J. Steen

**Affiliations:** Department of Dutch Studies, University of Amsterdam, Amsterdam, Netherlands

**Keywords:** metaphor structures, metaphor processing, deliberate metaphor use, utterance processing in discourse comprehension, fast and slow thinking, Deliberate Metaphor Theory

## Abstract

The immense increase in metaphor theory and research over the past decades is posing a threat of fragmentation to the field, which has been responded to by calls for new and more encompassing approaches to virtually all aspects metaphorical. This article argues that the opposite response may be more productive. By focusing on a different way of theorizing metaphor and its comprehension, existing theories and data can be re-ordered in an alternative and coherent way, which moreover breaks new grounds in tying up both with a general theory for all utterance comprehension as well as a general theory for all cognition as involving fast and slow thinking. The core of the new theory highlights the differentiation between deliberate and non-deliberate metaphor use, related to how people see the use of a metaphor as a metaphor in communication, that is, as a metaphor that counts as a metaphor between language users. It shows how this distinction can be employed to make sense of many insights about metaphor and its comprehension in innovative ways. The article outlines the foundations of the new theory and discusses how existing data, old and new, can be seen as supporting the new proposals.

## 1. Introduction

Since metaphor shifted from the humanities (philosophy, poetics, and rhetoric) to cognitive science (linguistics, psychology, and communication science), theories and hypotheses as well as evidence and data have exploded. The main outcome of this development today can be summed up in one word: variation. There is a daunting range of theories and hypotheses as well as a wealth of research and data that suggest that metaphor may not be one thing. Although many researchers accept that the use of metaphor is a form of understanding one thing in terms of something else, not all researchers do; nor do all manifestations of metaphor processing in the real world require conceptualizing one thing in order to understand another. There is arising a serious question of unity in diversity. As Semino and Demjén (2017: 2) write: “The sheer quantity, extraordinary variety and richness of recent work on metaphor means that the field can appear fragmented and overwhelming.” In some fundamental way, this is not just an appearance, however, but a confusing reality. It needs to be addressed head on.

Indeed, when the available data about metaphor structures and metaphor processes are combined, there may even be a paradox of metaphor, to the extent that, in language use and verbal discourse, most metaphor may not be processed metaphorically (Steen, 2008). This is a problematic situation that hints at another question: what would count as a real metaphor, or, more philosophically, what would be a paradigmatic case of metaphor? Upon close inspection, metaphor studies may be in a paradigm crisis, which does not facilitate fruitful discussion, let alone engender progress, between various schools of thought.

One attempt to address this situation for language use and discourse is Deliberate Metaphor Theory, or DMT (e.g., Steen, 2017, 2023). It aims at accounting for variation in the phenomenon and its theoretical modelling; it allows for considering new paradigmatic cases; and it may affect the definition of metaphor in language use and discourse. A new elaboration of DMT has presented its main argument in a more encompassing conceptualization and new operationalization (Steen, 2023), the core of which may be summarized as follows.

The cognitive-scientific paradigm has highlighted metaphor’s specific nature as a figure of thought as opposed to a figure of speech. However, research has shown that not all language use that can be seen as an expression of such figures of thought is comprehended by means of live metaphorical mappings between conceptual domains in on-going communication between people. Instead, only those figures of thought that are treated *as* metaphors by language producers and/or receivers, count as figures of thought in communication between language users and discourse participants, too. In advancing this proposal, DMT aims to resolve the paradox of metaphor. In DMT, all metaphor counting as metaphor in communication also gets comprehended metaphorically by means of some form of cross-domain mapping (or analogy), while all metaphor that does not count as metaphor in communication does not. In DMT, metaphor comprehension is hence not about figures of thought but about figures of thought that count as such in communication.

In addition, if all metaphor is a form of cognition, then it can also display different speeds of processing. Alluding to Kahneman (2011), DMT highlights that it is possible to think in fast and slow manners by metaphor, too. The idea that people can think by metaphor in slow manners, however, goes against the main trend in the cognitive-scientific paradigm. The new question concomitantly arises how this relates to the above-mentioned use of metaphor *as* metaphor in communication. This is addressed in the more recent elaboration of DMT (Steen, 2023) in such a way that it reinforces previous proposals of DMT and strengthens DMT’s position as a new approach to metaphor as a form of fast and slow thinking that lies at the heart of cognitive science.

This paper will discuss the present-day situation in metaphor studies as problematic because of the exponential increase in theoretical and empirical variation that is threatening to pull the field apart (Section 2). The proposed solution is to rethink the foundations of metaphor research in terms of DMT’s new theory and model for metaphor and its comprehension (Section 3). Section 4 will then consider old and new evidence that can be marshalled in favor of DMT. In the final discussion, the argument will be briefly reconsidered from the perspective of thinking by metaphor in fast and slow ways, and indicate some new prospects (Section 5).

## 2. The problem: variation in metaphor and its comprehension

### 2.1. Variation in theories and research

There are two sides to the immense variation of metaphor and its comprehension. On the one hand, an increasing amount of structure and process data is being generated by researchers, introducing ever new aspects of metaphor to the big picture. On the other hand, there is a substantial number of competing and partly overlapping theories of metaphor and its comprehension which account for different configurations of these aspects in partial models. A very brief inventory will help to suggest the scope of this variation, and provide a motivation for its alternative management in DMT.

The latest handbook of metaphor and thought opens with two sections, on the roots of metaphor and on metaphor understanding, offering no fewer than nine different theories and models (Gibbs, 2008). The new handbook on metaphor and language presents four theoretical approaches (Semino and Demjén, 2017), all of which are also covered by Gibbs (2008). In their review of models of figurative language comprehension, Gibbs and Colston (2012: 58–127) add several other models to the list of nine represented in Gibbs (2008). Overlap and competition are rife.

Most of these models have been sorted into three groups by Holyoak and Stamenković (2018), who review the state of the art in metaphor comprehension. They argue that there are three main strands in empirical research on metaphor processing, pointing to three distinct hypotheses:

1. Metaphor is processed by analogy.
2. Metaphor is processed by categorization.
3. Metaphor is processed by conceptual mapping.

According to Holyoak and Stamenković, the data collected and analyzed with reference to these hypotheses have not yielded one clear winner. There hence is not just a lot of variation in metaphor itself, but also in theories of metaphor processing; it is quite possible that these different theories cater to different aspects of variation in metaphor and its comprehension.

A recurring conclusion is that there is not just one process of metaphor comprehension and therefore not just one most successful model. Here are Gibbs and Colston (2012: 126) summarizing their review of models of figurative language comprehension:

We maintain that it is quite unlikely that certain default processes occur apart from contextual influences in each case of interpreting figurative meaning and that people do not always use contextual information in the same way in all discourse situations. The temporal course of figurative meaning interpretation depends on numerous factors that include the types of figurative language, the people involved, their likely goals and motivations, social and cultural context, local discourse interactions, and (quite importantly) the specific task used to study how language is processed and what is understood.

This is probably true. But it also assumes that the role of these “certain default processes” gets clarified. This is what DMT aims to do.

Gibbs and Colston’s (2012) view is completely in line with the conclusion drawn by Holyoak and Stamenković (2018), who also call for a more encompassing theory of metaphor comprehension. This should include a wider range of metaphors, pay greater attention to context and pragmatics, and develop connections with literary psychology. Ever new contexts are being examined and this increases the number of aspects of metaphor comprehension that need to be taken into account. The most encompassing version of this tendency is the proposal of a dynamical systems theory for metaphor (Gibbs, 2017).

As to variation in metaphor itself (as distinct from the process of metaphor comprehension and understanding), one focus of research is on the structures and functions of metaphor in language (as differentiated from metaphor in thought). Next to the section on theoretical approaches to metaphor and language, Semino and Demjén’s (2017) handbook has two sections devoted to formal variation and functional variation. Formal variation includes the role of the lexico-grammatical structure of metaphor, like the role of parts of speech, the combination of metaphor-related words into structural patterns in text, and the opposition between plain metaphor and simile. Formal variation is related to the contrast between novel and conventional metaphor in thought, which in language is often manifested as creative versus familiar, attested uses. Functional variation, by contrast, targets the role of metaphor in various domains of discourse, which constitute broad contexts for the use of metaphor in concrete utterances, including education, science, politics, advertising, and the internet. Yet another section in this handbook connects all this research to applied approaches to metaphor, which considers how metaphor is and can be utilized for problem solving in society: here the focus is on variation in metaphorical expression and conceptualization by topic, and the way in which this variation can be exploited in specific problem spaces, like private, professional, and public discourse about diseases such as cancer, HIV/AIDS, and dementia in health care. Structural-functional variation of metaphor in language use and discourse is great and many-faceted.

A comparable body of research on the structures and functions of metaphor in thought is not broadly available by itself. It is most often linked to work done by linguists and discourse analysts who approach the structures and functions of metaphor in thought via metaphor in language. Data focusing on the variation in the structures and functions of metaphor in thought by themselves can be found, however, in seminal theoretical publications on Conceptual Metaphor Theory (CMT), such as Kövecses (2015, 2020). Here, metaphor in thought in the form of conceptual metaphors is postulated to exist on four different levels of schematicity, ranging from image schemas through domains and frames to mental spaces (*cf.* Dancygier and Sweetser, 2014), and illustrations offer analyses of data that have often been constructed for the purpose by the analyst.

What is also important in this type of work is the variation in conventionalization of conceptual metaphors themselves, setting out from the simple opposition between conventional versus novel. Another source of variation at a conceptual level is metaphor aptness. And a third source of variation bears on the degrees of concreteness and abstractness of source and target domains. All of these are structural-functional aspects of metaphor in thought that play a role in the research reviewed by Holyoak and Stamenković (2018).

The common conclusion is that there is not just one structure and function of metaphor in language and in thought. It is true that, for metaphor in thought, one important representative of CMT does discern one kind of unity:

A major strength of CMT, and a source of its attractiveness, is that it offers new insight to a huge variety of topics and subject matters in the humanities and the social as well as the natural sciences. The insights all have to do with the fact that human beings are conceptualizing beings and that conceptualization, as suggested by CMT, is to a large degree metaphorical in nature. (Kövecses, 2021: 200)

This is a bold claim and points to an ambitious program about the structures and functions of metaphor in thought, which has been growing fast since Lakoff and Johnson (1980).

But the claim is not uncontroversial. For instance, Relevance Theory, offering an entirely alternative approach to metaphor in language, cognition, and communication, has labelled itself “a deflationary account” of metaphor (Sperber and Wilson, 2008), in order to signal disagreement with the inflationary account that is here represented by Kövecses (2021). In addition, Kintsch (2008) has argued that metaphor may be recognizable as a unified linguistic category, but that it does not exhibit one related psychological process. Kintsch’s position, in turn, however, is also problematic: to many linguists and discourse analysts, it is the very variation in metaphor as a linguistic category that is at issue and in need of one underlying conceptual basis (which accounts for the perceived dominance of CMT in research on metaphor in language, see Semino and Demjén, 2017: 8). The relation between metaphor in language and thought remains a great challenge.

These issues are often hotly debated, and this is the reason why Gibbs (2017) entitled his overview *Metaphor wars*. It is true that there is great interest in the potential role of metaphor, which may have exploded mostly as a result of CMT (Gibbs, 2017: 5–7); but it is much less clear what the status is of the insights generated by its empirical (as opposed to theoretical) investigation. Given this state of affairs, what reliable and valid knowledge has been produced about metaphor’s distinguishable structures, functions, processes, and effects? How can which data be interpreted best by which theories? This question is not easy to answer.

### 2.2. Problems in relating theory and research

The evidence for the wide range of available theories is, as might be expected, also rather varied and mixed. Indeed, different conceptualizations in different theories even should lead to different data, while null results for each of these theories do not commonly get published. If metaphor is not defined as understanding one thing in terms of something else (Lakoff and Johnson, 1980), but as a form of loose talk (Sperber and Wilson, 2008), then different sets of data will be deemed relevant. At the same time, they will also display overlap on account of their at least partly shared object of study.

Moreover, the varying popularity of distinct theories obviously affects how many and which types of data get produced. This creates the impression that there is massive evidence, for instance, for CMT, while there is much less evidence for a number of its competitors. Such a state of affairs is at least partly a reflection of the attractiveness of a theory and a hypothesis. Yet this does not mean that this theory and hypothesis are in fact better, or have been tested better by the data in comparison with the competition.

Variation in data counting as evidence is also due to the diverse nature and scope of the thousands of empirical studies that have been carried out to collect and analyze them. A case study in text analysis has a different value than a meta-analysis of dozens of experiments. Moreover, methods and techniques vary wildly between all of these publications, ranging from neuro-scientific research into the milliseconds of activation of parts of the brain to corpus work examining the distribution of specific sets of words across huge amounts of natural language use. More general conclusions may only be drawn in different and careful ways from all of these distinct studies. They may come together naturally in one encompassing framework like CMT, but this does not mean that they all contribute to one clearly formulated model of processes and structures. This in fact remains the negative conclusion of contemporary reviews, as we have seen. Frameworks, theories, and models are essentially different things.

Each of these methods and techniques do not only have their own strengths, but also their own limitations. The ecological validity of some psycholinguistic experiments has been contested, for instance, when it comes to using *A is (like) B* stimuli (e.g., Deignan, 2005; Gibbs, 2017): these forms hardly occur in natural language use, but have been the basis for much of the theoretical debate between competing approaches in psycholinguistics. In spite of this low ecological validity, however, the internal validity of these studies, and hence of their conclusions based on the particular experiment, may be great. The reverse may hold for other types of research. The problem for generating a more encompassing picture, both in terms of framework and model, lies in their generalizability to other types of materials and tasks.

Similarly problematic is the reliability of linguistic and conceptual metaphor identification and classification ‘in the wild’. In practice this depends on large degrees of shared but often implicit assumptions. These have only recently been made explicit for part of the question in new methods for metaphor identification in language use (Pragglejaz Group, 2007; Steen et al., 2010). The issue of reliable conceptual metaphor identification, however, or some equivalent outside CMT, is still open.

This holds even more forcefully for the postulated connection between a specific word or group of words, on the one hand, and a particular conceptual metaphor, on the other. This is a connection that typically has not been independently and systematically tested. More generally, the interpretation of structural-functional data of metaphor in language and discourse in terms of what they say about the associated processes of metaphor comprehension and understanding often involves leaps of faith. These may be highly informed and inspiring, but they still require independent investigation in behavioral research, which so far has kept offering a mixed picture.

Part of this situation has been addressed by the notion of converging evidence, which has been a cornerstone in the development of cognitive linguistics, the main basis for CMT (e.g., Schönefeld, 2011). Researchers have invested lots of attention in the relation between structural-functional, observational research versus processing-product, behavioral research in all kinds of areas of language use, including metaphor. This has especially worked as a way to motivate a practical division of labor between scientists coming from different disciplines, such as linguistics and psychology. But systematic attempts to investigate the same hypotheses, materials, and data about metaphor and its comprehension by means of these two broadly differing approaches are the exception, not the rule.

As a result, the evidence that is there, for distinct theories, can best be called piecemeal and local. There is a lot of data in specific studies, or groups of specific studies, which can be seen as valid and reliable evidence for theories about aspects of metaphor and its comprehension. Thus, there is growing corpus evidence about the distribution of metaphor-related words, for instance, in all sorts of situations of use. Functional interpretations of these patterns in terms of metaphorical conceptualization in cognition abound, but they need support and validation from process research on the effect of metaphor in comprehension and understanding and interaction.

Crucially, there is conflicting evidence about the role of analogy and cross-domain mapping in metaphor comprehension (Gibbs, 2017; Holyoak and Stamenković, 2018). This has even led to fundamental changes in the hypotheses involved in the theoretical debate: thus, according to Gibbs (2017; *cf.* Gibbs and Colston, 2012), it may be better to think of conceptual metaphors as emerging *from* comprehension, during understanding, instead of leading *to* comprehension, as was the bold original idea of CMT. The latter was the target of a lot of psycholinguistic research in the 1990s. If this view is now relativized if not abandoned, then the question arises: what does happen in comprehension? And when and how do conceptual metaphors emerge from comprehension, and in which forms?

All of this has also been noted by recent reviews, which have therefore called for new theories (Gibbs, 2017; Holyoak and Stamenković, 2018). There has even been a call for a preliminary consideration of the criteria for such new theories (Zlatev et al., 2021). This is not because there is a generally accepted clear and trustworthy picture of metaphor and its comprehension.

### 2.3. The need for a new model: bigger or smaller?

Consider the following quotation from the online *Stanford Encyclopedia of Philosophy* entry on Thomas Kuhn by Alexander Bird (https://plato.stanford.edu/entires/thomas-kuhn, last accessed April 6, 2023):

Kuhn describes an immature science, in what he sometimes calls its ‘pre-paradigm’ period, as lacking consensus. Competing schools of thought possess differing procedures, theories, even metaphysical presuppositions. Consequently there is little opportunity for collective progress. Even localized progress by a particular school is made difficult, since much intellectual energy is put into arguing over the fundamentals with other schools instead of developing a research tradition. However, progress is not impossible, and one school may make a breakthrough whereby the shared problems of the competing schools are solved in a particularly impressive fashion. This success draws away adherents from the other schools, and a widespread consensus is formed around the new puzzle-solutions.

Bird’s summary of a pre-paradigmatic period in some scientific field seems to be applicable quite nicely to the situation in metaphor studies. In that picture, CMT may have been the breakthrough “whereby shared problems of the competing schools are solved in a particularly impressive fashion.” CMT has indeed generated widespread consensus around the new puzzle-solutions, especially in the structural-functional study of metaphor in language and thought. This has also generated a whole new set of paradigmatic examples of metaphor and how it is processed in use.

However, the borders of this solution now may have been reached. Variation and sometimes fragmentation appear to take over from unity in occasionally bewildering ways. This is leading to the above-mentioned call for new theories that can encompass even more phenomena, both in terms of structure (and function) as well as psychological process (and product). But much more than a call for new developments in this direction, and indications of what has to be included in such a new research program, is not really there.

There is an alternative solution. We should focus on one of the key problems in the debate between the various schools and address this problem in more radical terms than has been done so far by the existing schools. This would yield a theory that attempts to clarify the relation between hypotheses and data on the basis of what has been learned by 40 years of speculation and research.

A radical way of responding to the growing attention to variation in metaphor and its processing is to adopt the following position: “not all metaphor in language and thought apparently works as metaphor in communication.” This claim is a new way of handling the paradox of metaphor (Steen, 2008), which says that not all metaphor in the structures of language and thought may be processed metaphorically, that is, by means of a local analogy or more extended conceptual cross-domain mapping (*cf.* Holyoak and Stamenković, 2018). The paradox is an embarrassing problem for many metaphor researchers, which is caused by the limitation of their view to metaphor in language and thought.

The paradox can be resolved when the perspective is broadened beyond metaphor in language and thought to metaphor in communication. The main hypothesis is that, for language use and discourse, there is an interaction between metaphor and communication. In particular, when metaphor counts as a metaphor in communication, it is comprehended by analogy or cross-domain mapping, and when it does not count as such, it is comprehended by categorization and lexical disambiguation. This conceptualization of the interaction between metaphor and communication resolves the paradox of metaphor by claiming that all metaphor in communication is comprehended metaphorically, while all metaphor (in language and thought) that does not count as metaphor in communication is not comprehended metaphorically.

The move to include communication as another dimension besides thought and language in the model for metaphor and its comprehension has been around explicitly in Relevance Theory and less explicitly in discourse approaches to metaphor. There is no doubt that metaphor research has profited from these approaches. However, they have not resolved the paradox of metaphor and have not led to agreement about a greater unification of the field. Indeed, Relevance Theory has greatly downplayed metaphor as a category of its own. It would be productive if all of these tendencies could be brought together in one explicit framework, theory, and model, and this is what DMT hopes to facilitate.

The next section will sketch out the foundations of this new theory. Data that can be marshalled in its support will be provided in Section 4. The new approach offered by DMT is also important for the impact metaphor studies can have in other domains of research as well as in society, which will be served by broadening the framework of metaphor studies in Section 5 to slow thinking by metaphor.

## 3. The solution: rethinking metaphor comprehension

### 3.1. Four dimensions of metaphor in utterance representation in discourse comprehension

DMT hypothesizes that there is a fundamental processing difference between metaphor when it is used *as* a metaphor in communication, on the one hand, for instance as in the case of a simile, versus metaphor not used as a metaphor in communication, on the other, for instance as with the regular temporal and abstract uses of spatial prepositions (Steen, 2008, 2023). This is another type of variation, which can be added to the many different types of variations already observed. It can be associated with readily distinguished structural-functional categories of variation such as metaphor versus simile, and novel versus conventional metaphor (*cf.* Semino and Demjén, 2017). And it can be based in specific hypotheses and data in comprehension research involving categorization (plus lexical disambiguation) versus analogy (plus cross-domain mapping) (*cf.* Holyoak and Stamenković, 2018). This is where DMT sets out from existing hypotheses and data.

Yet DMT requires a new model for metaphor comprehension that goes beyond the currently dominant two dimensions of metaphor in language and thought (including, respectively, metaphor versus simile, and novel versus conventional metaphor), a status quo which also has limiting consequences for what counts as context. Contrary to what seems to be a widely shared assumption now, communication in the real world is not a context for individual metaphor comprehension, but an integral part of it, as is also stressed in Relevance Theory (see also Soares da Silva, 2021). This is a less common view, which is afforded, however, by grounding metaphor comprehension in a more general and widely accepted multi-dimensional model for all utterance comprehension in discourse. This is the Construction-Integration model developed by Walter Kintsch and Teun van Dijk over the past decades (Kintsch and van Dijk, 1978; Van Dijk and Kintsch, 1983; Kintsch, 1998; Van Dijk, 2008). It models utterance comprehension in its communicative relation to the text in the discourse situation and its users, and it has had a great impact on all discourse comprehension theory and research (e.g., Pickering and Garrod, 2004, 2013; *cf.* McNamara and Magliano, 2009; Schober et al., 2018).

DMT’s use of this model shows roughly the following conditions for metaphor comprehension (Steen, 2023: 115–136). When language users comprehend an utterance in a discourse event, they construct four distinct but related mental models for the meaning of the utterance. These mental models yield a surface text (representing aspects of language), a text base (representing aspects of thought), a situation model (representing aspects of the microworld referred to by the utterance), and a context model (representing communicative aspects of the utterance in the discourse event). The content of these four mental models naturally exhibits which aspects of meaning of a metaphor are predicted to be relevant for the generation of an appropriate utterance meaning in discourse comprehension. This produces a four-dimensional model for metaphor in utterance processing in discourse, which we will return to in Section 3.2.

DMT has advanced an associated 4D model for the structures and functions of metaphor itself as well. It argues that metaphor does not just have linguistic and conceptual properties, including metaphor versus simile in language and conventional versus novel mapping in thought, but that metaphor also exhibits referential and communicative properties, notably the difference between direct and indirect reference to the source domain, and deliberate versus non-deliberate use in communication. This is hence an elaboration of the original 3D model called for in Steen (2008) as a result of on-going research (Steen, 2023): the dimension of reference in the situation model has now been distinguished from the other three dimensions that were postulated before.

For metaphor, the crucial question for reference and for communication is this: Is an utterance meant to make the addressee really think of one thing in terms of something else, or is it not? Or, in terms of the addressee: Does the addresse see the utterance as meant to make them understand one thing in terms of something else, or not? In other words, is it part of the (communicative) meaning of the utterance that it involves making a comparison between two distinct things that are both somehow involved in the meaning of the utterance? These questions go to the heart of how metaphor works.

This is why the new slogan of DMT is: “to compare or not to compare, that is the question”. This clearly is a very different solution to the paradox of metaphor than what has been proposed in Relevance Theory, which sees metaphor as a form of loose talk without requiring comparison, although similar developments can be discerned in Carston (2010, 2020; *cf.* Carston and Wearing, 2011). What is more, this approach also allows for people’s finding and resolving figurative comparisons in much less fast and automated ways than has been the typical case in modern metaphor studies (Steen, 2023; *cf.* Kahneman, 2011). Instead, some metaphor comprehension is in fact much closer to problem solving than to fast and automated language use (Kintsch, 2008).

Here is one new and radically surprising outcome of this approach (Steen, 2023). We know that the bulk of metaphor is indirect and conventional (Steen et al., 2010): allusion to the source domain is indirect, and the source domain itself is a widely attested form of conceptualization for the target domain. This is because most metaphor is based in lexical polysemy, as has been a corner stone of present-day metaphor research. As a result, however, utterances that contain this kind of metaphor are in fact potentially ambiguous between two readings. They can be seen as either deliberately metaphorical, requiring some form of active comparison (or, figurative analogy), or as non-deliberately metaphorical, which does not require comparison or analogy for the intended utterance meaning. This ambiguity has been missed in most metaphor research, but its recent discovery in DMT (Steen, 2023) has big consequences for the status of these metaphors as metaphors in language use and discourse.

Consider, for example, *She died yesterday after a long fight against cancer* (*cf.* Steen, 2023: 163–168, 278–282, 298–317). In the deliberate reading, this utterance is intended to invoke a live cross-domain mapping, which requires comparison or figurative analogy between the source domain of fighting and the target domain of a determined effort to prevent something from turning bad. In the non-deliberate reading, this utterance is intended to talk about cancer in terms that are familiar for cancer discourse, without invoking any cross-domain comparison. This reading does not require analogical comparison, but can be handled by lexical disambiguation (e.g., Gentner and Bowdle, 2008; Giora, 2008; Glucksberg, 2008; Kintsch, 2008). The surprising point is, though, that, other things being equal, both types of readings are possible. It is the intentions of the sender, then, but also, and even independently, of the addressee that decide which reading (based on which interpretation of *fight*) is deemed most relevant in comprehension. This does not even have to lead to the same result, which can cause a problem for the interaction and its continuation.

The referential and communicative meanings of the utterance change accordingly. In the deliberate reading, their combination can be paraphrased like this:

“The speaker means to say that she died yesterday after a long (literal) fight against cancer (where a (literal) fight with an enemy is meant to be similar to a determined attempt to stop cancer)”.

In the non-deliberate meaning, it can be paraphrased like this:

“The speaker means to say that she died yesterday after a long determined attempt to stop cancer”.

These are two diverging meanings. The one involves thinking by metaphor, while the other does not. They can be formally expressed and seen as predictions for the two versions of possible content of people’s mental representations of the utterance during comprehension of this utterance (Steen, 2023: 280, 314–315).

Language users who are critical of violence metaphors for talking about cancer (*cf.* Semino et al., 2018; Wackers et al., 2021) may legitimately comprehend the utterance as involving a live metaphor, and spell out the first reading of the utterance as what they hear was conveyed and then raise their objections. But language users who do not intend to talk about cancer in terms of violence but simply wish to use the familiar, conventional linguistic means at their disposal, may legitimately deny that they intended to convey such a comparison. They may point to a user’s dictionary such as Macmillan and argue that the word *fight* simply and conventionally means “a determined attempt to stop something.” They may also argue that that was all they thought of when they expressed the utterance. And this may clearly also be what many hearers will understand, and, indeed, what a language learner may want to learn about the meaning of the English noun *fight*. That linguists and critical language users may point to connections between the conventionalized figurative meaning and the (perhaps original) non-figurative meaning, is another matter.

As a result, we have two communicative scenarios that, in principle, are equally possible, with one scenario requiring analogical processing, or thinking by metaphor, while the other does not. Moreover, we also see that either scenario is crucially linked to language user intentions, both for production and for comprehension. What is more, the one scenario, with the analogy, in fact also sets up a form of reference of its own to the source domain of fighting, or physical violence, as part of the meaning of the utterance. The second scenario does not do so, and the source domain of fighting or physical violence is not part of the projected state of affairs about handling the disease of cancer. There is a difference between metaphor in language and thought (where we do have the metaphor-related word *fight* in the surface text and its related concept fight in the text base), on the one hand, and metaphor in reference and communication on the other (where we have two different states of affairs in the situation model, and two different communicative intentions in the context model). This illustrates how there are four different dimensions in comprehension that can convey different meanings and suggest different cognitive processes for the complex comprehension of metaphor.

### 3.2. Framework, theory, and model

The updated version of DMT starts out from a theoretical framework that includes several big factors of metaphor use (Steen, 2023). These are distinct activities of cognition (e.g., comprehension and production), levels of cognition (language use and discourse), cognitive processes (lexical disambiguation, categorization, analogy, cross-domain mapping, and comparison), and speeds of cognition (thinking fast and slow, a little less fast, and even slower). This framework can be seen as an overall ordering of bigger factors and more distinct variables, which any theory of metaphor needs to adopt some position about. There probably are more than just these four, as is also suggested by the calls for encompassing theories and their preliminary criteria mentioned above. DMT’s selection makes a start, is compatible with most theoretical and empirical insights, and is already quite complex.

DMT then proposes its own theory of metaphor and its comprehension, which sets out from a general processing model for utterance comprehension, the Construction-Integration model. The CI model itself is based on a more encompassing theory of all utterance processing in discourse, and this kind of theory is what any theory of metaphor in language use and discourse should eventually be compatible with. The theoretical framework of the CI model therefore also provides a theoretical framework for re-thinking the model for metaphor comprehension. This is particularly important for distinguishing the dimensions of language and thought as well as reference and communication in terms of processing (the four mental models) as well as metaphor structures and functions (the four dimensions of metaphor).

In particular DMT proposes that, for language use and discourse, the key hypothesis about metaphor comprehension turns on deliberate versus non-deliberate use. This is primarily linked to the dimension of communication and the cognitive process of building a context model. DMT claims that language users somehow decide whether a metaphor is intended as a metaphor in communication, that is, counting as a genuine metaphor between language users, or not. This happens in production and comprehension (activities), whether a metaphor is expressed within the boundaries of an utterance or across utterances as distinct units of discourse (levels), and whether this occurs fast and automatically or slow and in a more voluntary way (speeds).

The crux of this hypothesis turns on the fourth remaining factor of the above framework: processes. DMT claims that deliberate metaphor use always requires processing by analogy (or its more extended manifestation of cross-domain mapping) and therefore also involves comparison. This is live understanding of one thing in terms of something else. Here metaphor is being comprehended metaphorically.

Non-deliberate metaphor, by contrast, does not require processing by analogy. Instead, it is handled by lexical disambiguation and categorization. This is not live understanding one thing in terms of something else. But it is also true that relations between a word’s senses and its related concepts can be interpreted as involving understanding one thing in terms of something else, and this may happen in post-comprehension understanding.

In the DMT model for metaphor comprehension, people build four representations in a row, even though this clearly is a simplification: for the complexities of a full computational model, see Lemaire et al. (2006). In this initial, more simple model of processing, language users in comprehension first build a representation of the utterance as part of a surface text, and this is where they represent its linguistic features; they then build a representation of the utterance as part of a text base, where they represent its conceptual features; next, the text base is used to project a situation model, which captures the picture of the world evoked by the utterance in terms of its referential properties; and finally they include this situation model in a more encompassing context model that represents how the situation in the utterance plays a role in what the sender wants to communicate to the receiver. Again, the step-by-step ordering of each complete mental model is a simplification: it helps theory development and presentation, but in practice a more complicated model is needed that roughly works on word-by-word and phrase-by-phrase basis (Lemaire et al., 2006).

The crucial transition in the model occurs when the Construction stage (building surface text and text base) moves into the Integration stage (constructing a situation model and context model). DMT holds that for non-deliberate metaphor use, lexical and conceptual disambiguation discard with source-domain senses and related concepts, and that the situation model as a result will only exhibit a target-domain referent. To illustrate with the help of our example of *She died yesterday after a long fight against cancer*, DMT’s application of the CI model predicts the following non-deliberate scenario:

1. In the surface text, the word *fight* is represented as polysemous between a physical fight and the more abstract fight of a determined attempt.
2. In the text base, both of these senses activate related (sub) concepts for fight, which contribute to the building of propositions for a text base and enable the projection of a situation model.
3. In the situation model, the situation that is not metaphorical is in this case preferred, yielding a state of affairs that has the following referential structure: ‘she died yesterday after a long determined attempt to stop cancer’.
4. In the context model, this situation model is included in a representation of the communicative intentions of the sender: ‘The speaker means to say that she died yesterday after a long determined attempt to stop cancer’.

This analysis of the utterance is automatically produced by the comprehension model, predicts what language users will do when they receive this utterance as a non-deliberate metaphor, and at the same time offers a structural-functional description of its meaning.

Compare this with the situation when the metaphor is used deliberately. The first two steps are identical:

1. In the surface text, the word *fight* is represented as polysemous between a physical fight and the more abstract fight of a determined attempt.
2. In the text base, both of these senses activate related (sub) concepts for fight, which contribute to the building of propositions for a text base and project a situation model.
3. In the situation model, however, fight is used to project a referent that involves physical violence against some opponent: ‘she died yesterday after a long physical and violent fight against cancer.’ Since people as a rule do not physically and violently fight with a disease, this creates a problem of coherence. DMT posits that it is this coherence problem that triggers the recruitment of analogy or cross-domain mapping as a problem solving device (*cf.* Miller, 1979). This move involves setting up an extra step to create a new situation model that referentially is coherent and therefore does make sense: ‘she died yesterday after a long (literal) fight against cancer (where a fight with the enemy is similar to a determined attempt to stop cancer)’.
4. The adjusted situation model is finally incorporated within the context model: ‘The speaker means to say that she died yesterday after a long (literal) fight against cancer (where a fight with the enemy is similar to a determined attempt to stop cancer)’.

In this way, DMT accounts for the contribution of the metaphor-related word *fight* to the ambiguous meaning of the utterance and the way that it is predicted to be processed in comprehension in two different ways: as non-deliberately metaphorical and as deliberately metaphorical. In principle, these two interpretations are equally possible, the utterance is ambiguous between deliberate and non-deliberate use, and this is one novel finding of DMT (Steen, 2023).

The new question that this reveals is: how do we know whether an utterance is meant to be deliberately metaphorical or not? In other words, how do we know whether metaphor-related words count as a metaphor in communication in an utterance in a specific discourse event? This also includes the question, for which specific language user, or class of language users, and discourse participant(s), and in which activities (production, reception, interaction)? This is hence where other theoretical components come into play.

### 3.3. Deliberate versus non-deliberate metaphor use in comprehension

The central hypothesis in DMT generates a number of subsidiary hypotheses and specific predictions. The structures and functions of metaphor are one crucial factor for suggesting whether a metaphor can be used deliberately. They are closely linked to experimental research on whether a particular class of metaphors is indeed processed by analogy. Structures and functions are not the only factor, but they clearly provide a baseline. There are three main structures that have been shown to be crucial by now: presence or absence of metaphor signaling, conventionality versus novelty, and (in)directness.

Signaled metaphors, novel metaphors, and direct metaphors are all metaphor structures that promote deliberate metaphor use. Thus, the preposition *like* signals the intended need for comparison, whether this is figurative or not. Novel metaphors do not have a conventionalized target domain and the sender of the utterance commonly intends that this has to be constructed on the spot by analogizing from the novel source-domain element; if this does not happen, the novel source-domain element would present an incoherent relation with the utterance and the discourse. Direct metaphors are metaphors that (intentionally) present a direct expression of one or more elements of some source domain, and these need to be integrated within the surrounding target domain by means of analogy, too. The relations between these metaphor structures and the deliberate use of a metaphor has been the basis of DMT’s predictions from the start.

The role of these properties can now also be explained by the 4D model. Thus, metaphor signals are part of the linguistic dimension of the model; they are represented in the surface text. Novel versus conventional metaphor is part of the conceptual dimension of the model; relevant properties emerge in the text base. And direct versus indirect reference to the source domain is part of the referential dimension of the model; it can be seen as part of the situation model. These structures relate to the intention to use a metaphor as a metaphor or not, which is part of the communicative dimension, and is handled in the context model. This systematic approach afforded by DMT’s employment of the CI model hence further grounds these aspects of metaphor within DMT as a theory, aspects which have received a lot of attention in general metaphor research.

By contrast, non-signaled, conventional, and indirect metaphors do not promote deliberate metaphor use. They are more associated with non-deliberate metaphor use. Given the possible implications of the ambiguity of the *fight* example above, however, this view may have to be nuanced. Perhaps many if not most indirect conventional metaphors are structurally ambiguous between deliberate and non-deliberate use. It may therefore be their interaction with aspects of the discourse situation that decides how the ambiguity is resolved in actual use by promoting a decision about intentions on the part of the language user. These aspects of the discourse situation obviously include a metaphor’s discourse purpose as well as the discourse domain of the discourse event and characteristics of the participants involved in it.

DMT predicts that all of this automatically leads to a representation of some metaphors as intended as metaphorical, or deliberate, in the context model. By contrast, for most metaphors it does not, and these count as non-deliberate metaphors. This is the core proposition formulated by DMT and a decade of research is now beginning to outline the main trends (Steen, 2023).

Why would most metaphors not be deliberate? Here the shift of attention in DMT from language and thought to communication and reference can help. In retrospect, the emphasis on language and thought in early cognitive-scientific work on metaphor focused on one important function, which is conceptualization and its verbal expression. The shift of attention to communication (and reference) reveals another important function: perspectivization (and its relation to mental world-making). In language use and discourse, metaphors most often serve just for conceptualization and expression, exhibiting specific and situated uses of conceptual and linguistic systems, as has been shown in four decades of research. But what happens in language use and discourse in terms of communication is something essentially different: there we can observe the use of metaphor for offering an alien perspective on some target referent and topic, in order to do a comparison between two unlike things that are each referred to as distinct elements in the situation model.

In general, people do not go around making live comparisons as parts of utterances and texts all the time. This includes making metaphorical comparisons. We just use language and discourse for thinking and talking about things in the concepts and terms that are part of our conceptual and linguistic systems. That some of them are linguistically and conceptually metaphorical is irrelevant for the communicative side of those acts of language use and discourse events. When these metaphor-motivated aspects are there, metaphor-related words and concepts are also present in the surface text and text base as forms of polysemy, but, and this is crucial, they are absent from the situation model and context model (Steen, 2023). This is how DMT models the workings of these different classes of metaphor in specific situations of use, where potential ambiguity between deliberate versus non-deliberate metaphor use is resolved by the intentions of the specific language user and discourse participant whose comprehension process is being modeled.

The model can be further refined by the proposals advanced by Cuccio (2018). Her analysis of neuro-scientific research on metaphor suggests that, for conventional metaphor, all relevant senses and sub concepts may be in play during the Construction stage of lexical access and concept activation [as is also suggested by Giora’s (2008) Graded Salience Hypothesis]. This spread of automatic and ubiquitous sense and concept activation would lead to automatic attempts of concept combination, too, for the purpose of constructing a text base that can ground the projection of a situation model (*cf.* Kintsch, 1998). This blind combining process may also include neuro-cognitive activation of source-domain concepts for the text base. This can even be interpreted as involving a form of metaphoric thought.

However, when during the beginning of the Integration stage preference is awarded to those concepts and referents that exhibit conventionalized target-domain meanings, metaphoric thought is cut short. Then the utterance gets interpreted and represented as non-deliberately metaphorical. It sets up situation models that are restricted to the target domain, and this is what is the result of lexical and conceptual disambiguation for the purpose of integration. This situation model and context model consequently do not manifest metaphorical thinking. DMT adopts this subsidiary hypothesis about early source-domain activation and subsequent abandonment as entirely compatible with its theory and model.

This process can even be influenced by another variable, which is the varying salience of the distinct word senses that get activated in lexical access (Giora, 2008). Sometimes figurative senses are stronger and get activated faster than non-figurative (original source-domain) senses. This would increase their chances of forcing a non-deliberate interpretation of the metaphor, and would promote their processing via lexical disambiguation. The intended target-domain interpretation of the polysemous word is then readily available and first accessible and, other things being equal, this would be the meaning of the utterance that would count as adequate and relevant.

Finally, this model of metaphor comprehension is also compatible with the potential emergence of conceptual metaphor during full metaphor understanding, after a metaphor has been sufficiently comprehended (Gibbs, 2017). At that point, further processing of source-domain aspects may set in during processes of interpretation. They may even pick up from what was left behind before, as is also suggested by Giora (2008). In this way, DMT can offer specific roles to previous theoretical proposals as reinterpreted within one encompassing and explicit model.

### 3.4. Language use and discourse

The next step in modelling deliberate versus non-deliberate metaphor use in comprehension has to do with the interaction between metaphor structures and functions on the one hand and the factor of level of cognition on the other (*cf.* Deignan et al., 2013). This suggests that metaphors expressed as structures and functions at the level of discourse (e.g., Musolff, 2004, 2016a; Charteris-Black, 2011; Ritchie, 2017) may be distinct from metaphors expressed at the level of language use (e.g., Semino et al., 2018). In the former case, a cross-domain mapping is expressed between several utterances, while in the latter, it stays within the limits of one utterance. In the former case, metaphors work as structural-functional elements of text-in-code-in-context, in the latter, as structural-functional elements of utterances (Steen, 2023). Metaphors within utterances are probably the paradigmatic case of metaphor today.

DMT holds that metaphors expressed at the level of discourse work in a way that is typical only of that class. This is because they are always used deliberately as comparisons between distinctly expressed, unlike entities that work across utterances. They hence always require processing by analogy or cross-domain mapping. They are hence also the most extensive and therefore prominent example of genuine thinking by metaphor. This also more easily allows for slowing metaphor down (Steen, 2023).

An extended comparison, continuing a deliberate metaphor in one utterance through one or more following utterances, is not only deliberate at the level of language use (in the separate utterances) but also deliberate as a whole (in the text). It requires planning and processing as a whole, and is constitutive of (a substantial part of) the text. A well-known example is Shakespeare’s Sonnet 18: lines 1 through 12 build a concrete and specific comparison between the speaker’s lover and a summer’s day that is more specific than line 1 by itself.

Metaphor expressed at the level of discourse is a matter of secondary modeling (Steen, 2023), which has to do with building a text as a story, an argument, an exposition, and so on. Secondary modeling makes use of utterances, that in themselves manifest primary modeling, involving a state, a process, and action, and so on. Primary modeling yields states of affairs, secondary modeling yields text types. Primary models also have different functions than secondary models: utterances have speech act functions like promising and directing, while secondary models have discourse functions like informing, persuading, and instructing. Metaphors within utterances can be combined to build a metaphor between utterances that requires its own contents in the mind. This is what is organized by the factor of level.

Another type of case would be texts having a paragraph in the language of a source domain which is then meant to be compared with another, roughly equivalent or closely related, paragraph about the target. This is also clearly deliberate. An atypical example is a TED-talk about sex as eating pizza (Steen, 2023, chapter 8). In that talk, Al Vernacchio discusses how aspects of baseball are relevant to talking about sex without Vernacchio mentioning sex explicitly in that paragraph; he then moves over to do the same for eating pizza in the next paragraph, moves back to another aspect of baseball in the following paragraph, and then returns to eating pizza in the fourth paragraph. The language and discourse ostensibly are just about baseball, eating pizza, baseball, and eating pizza. But the meaning of the talk is about baseball in comparison with sex, eating pizza in comparison with sex, and so on. This comparative intention is explicitly announced at the beginning of the text. This is highly deliberate metaphorical meaning, and it requires hard thinking by metaphor, both in production and in reception. In the event of the talk, speed of cognition is controlled by the speaker, but when you see the film of the TED-talk, or read the transcript, then you can slow down according to your own needs and interests.

Other well-known examples concern even more encompassing stretches of text that are supposed to function as source domains for an explicit or implicit target, such as parables and allegories (*cf.* Ritchie, 2017). A famous example is George Orwell’s *Animal Farm.* And this situation does not just apply to narrative texts, but also to argumentative and expository texts. The hypothesis is that all of these are deliberate metaphors expressed at the level of the discourse event (as differentiated from language use), typically because parts of the source domain are directly expressed as distinct parts of the text. What this means in practice is that they all require analogical processing of their structures and functions. This may also be the place where thinking by metaphor is more easily or frequently slowed down.

All of the source-domain elements (summer’s day, baseball, eating pizza) that function as part of such a cross-domain mapping at the level of the discourse are part of a deliberate figurative comparison that enfolds in the growing situation model which includes them as referents in the content of the discourse. They directly refer to the source domain as one leg of the comparison. This must therefore be deliberate, that is, intended by the producer of the text. There is no ambiguity here between a deliberate and a non-deliberate reading, as is possible within the confines of one utterance for conventional indirect metaphor. This basis in extended source-domain reference would be a working hypothesis for the reason why metaphors expressed at the discourse level may always be deliberate and require analogical thinking that occasionally takes a lot of cognitive effort and time.

This view has knock-on effects on DMT’s view of another type of metaphor that is quite well-known too. This is when two or more sentences exhibit vocabulary that derives from one particular semantic field that is conventionally used to talk about a topic related to another semantic field, for instance cancer as violence or war. The consistent presence of such violence or war vocabulary across utterances is often taken as a reflection of the fact that senders really think about one thing in terms of something else, and that addressees need to do so too if they want to catch their meaning (e.g., Semino et al., 2018). This would thus be another type of metaphor expressed at the level of discourse that needs to be described and explained.

DMT has a different perspective, and argues that this may only hold throughout all utterances if one of the expressions at some point is clearly deliberate, for instance in the form of an explicit comparison. If this does not hold, then this manifestation of metaphor is similarly ambiguous between deliberate and non-deliberate use as our *fight* example above. In that case it is also possible, and even quite plausible, that all metaphor-related words are used as non-deliberately metaphorical, or, simply the way to talk about a particular topic. This would mean that neither in production nor in comprehension any of these words would necessarily activate a cross-domain mapping, since they can be handled by lexical disambiguation within the boundaries of distinct consecutive utterances. This especially holds if the source-domain meanings pertaining to for instance violence and war are less salient than the conventionalized target-domain meanings pertaining to handling a serious or difficult situation. These are testable predictions.

In production, such series of words may arise by lexical priming and by the conventional way to talk about a topic. The selected words therefore do not have to come from some scenario that would be in the mind of the producer. This too can work simply from one utterance to the next without requiring the presence of a metaphorical plan for the structures and functions of the discourse. For DMT, the situation changes if the relevant scenario actually comes up in the referential dimension as well. If the relevant source-domain concepts are discarded during the move from the text base to the situation model, however, then it is only the related and conventionalized target-domain equivalents that go through to the situation model, as is probably the case with most indirect conventional metaphor. DMT hence predicts that the postulated kind of framing effect of consistent series of metaphor-related words and their possibly associated scenarios is minimal, since they are typically indirect and conventional and therefore promote non-deliberate use within the utterance.

This issue is intimately connected with the question of the purpose of a metaphor, which has come to be seen as the role of a metaphor in the discourse event it is playing (Charteris-Black, 2012; Semino et al., 2018). As noted above, metaphors generally have the function of conceptualization or perspectivization in language use, which is one level of cognition, but this in itself can also have a purpose in the discourse event it is in, which is another level of cognition. DMT posits that non-deliberate metaphors do not have discourse purposes, because they do not count as metaphors in the communicative dimension of language use, and therefore not in the discourse. They essentially disappear from the mental representations of situation model and context model during the integration stage of comprehension. Deliberate metaphors, by contrast, can also have discourse purposes: their construction of an intended local or more extended comparison is often clearly done for a purpose, which can be related to several aspects of a discourse event. For instance, deliberate metaphors can function as various parts of setting up an argument (e.g., Renardel de Lavalette et al., 2019; Van Poppel, 2020; Finsen et al., 2021; Wackers et al., 2021). People can then legitimately wonder why a particular alien perspective in one or more utterances is used by the producer of some argumentative or other text.

In all, then, DMT has a number of theoretical proposals about the role of deliberate versus non-deliberate metaphor use in language use and discourse. They are based on a combination of theories of metaphor comprehension, the four-dimensional CI model of all utterance processing in discourse comprehension, and the various structures and functions of metaphor that have been uncovered in language use and discourse in the past four decades of metaphor research. It is now high time to turn to the way in which these hypotheses can be related to old and new data.

## 4. From theory to research: old and new evidence

### 4.1. Data before DMT

Data for examining the hypothesis that there is an interaction between metaphor structures and metaphor processes were first collected in order to test the Career of Metaphor Theory (Bowdle and Gentner, 2005; *cf.* Gentner and Bowdle, 2008). This proposal was a response to the then state of the art and the resulting contrast between the comparison versus the categorization views. This is another way of distinguishing between the three positions differentiated by Holyoak and Stamenković (2018), who also discuss the wide range of data supporting each of these positions. Given the availability of this review, explicitly repeating the data from all relevant studies here is not necessary, but they include reaction time studies, metaphor interpretation tasks, metaphor rating studies, neuro-imaging studies, and so on. The overall picture suggests that categorization, comparison, and conceptual mapping may each play a role depending on different linguistic and conceptual structures. DMT has brought this together in one coherent theory and model that expands the Career of Metaphor Theory.

The Career of Metaphor Theory claims that there are two main sets of data (in favor of lexical disambiguation plus categorization versus analogy plus comparison) and that their related hypotheses are valid, but for different sets of cases, and that this is due to the evolutionary status of the conceptual structure of a metaphor. Moreover, the Career of Metaphor Theory also claims that there is an association between this evolution and the preferred ways of expressing a metaphor in distinct linguistic forms, such as plain metaphor versus simile. According to the Grammatical Concordance Hypothesis, conventional metaphors associate more with categorization (and its expression as plain metaphor), while novel metaphors associate more with comparison (and its expression as simile).

The data in Bowdle and Gentner (2005) were collected because previous studies had shown that conventional metaphors were comprehended faster and differently than novel metaphors, which was taken as a reflection of the assumption that conventional metaphors have highly accessible metaphorical meanings. Therefore Bowdle and Gentner (2005) collected preference ratings that showed that conventional metaphors were more compatible with categorization statements than with comparison statements, which agrees with the idea that novel metaphors still work as comparisons while conventional metaphors do not necessarily do so. Thus, taking examples from Bowdle and Gentner’s materials, for conventional metaphors *Faith is (like) an anchor* or *A soldier is (like) a pawn*, the preference for the categorization form (that is, without the preposition *like*) was higher than it was for novel metaphors such as *A mind is (like) a kitchen* or *A beach is (like) a grill.*

In a second experiment, the same stimuli were offered for comprehension. Novel metaphors were comprehended more slowly than conventional metaphors. In addition, there was the predicted interaction with linguistic form: when novel metaphors were offered as similes they were comprehended faster than when offered as metaphors; conventional metaphors, by contrast, were comprehended faster when offered as metaphors than as similes. All of this is in accordance with the Career of Metaphor Hypothesis and the Grammatical Concordance Hypothesis. It became the basis of DMT (*cf.* Steen, 2008).

But DMT offers new interpretations of these existing data. First, the focus on simile (versus metaphor) brings out two dimensions out of four in DMT: in the 4D CI-model, simile includes both linguistic form (*like*) as well as direct reference (the word expressing the source-domain concept). Second, Glucksberg’s (2008) original hypothesis as well as data in favor of processing by categorization included a notion of “dual reference”, to both source-domain and target-domain aspects of meaning of the metaphorically used word. This notion cannot only be related to the ubiquity of polysemy, as was done by Bowdle and Gentner (2005: 198), which is a matter of the dimension of language; but it can now also be related to what is a genuine potential for dual reference in the situation model, as in the example *She died after a long fight against cancer*. And third, the expression of conventional metaphors as similes (instead of metaphors) may precisely lead to their revitalization as deliberately figurative instead of their use as non-deliberately metaphorical; the need for constructing a live analogy or cross-domain mapping because of the simile form, in spite of the conceptual conventionality, may precisely be the reason why these take longer to process.

Evidence related to comprehending metaphor-motivated polysemy and its handling by lexical disambiguation was collected in research for Rachel Giora’s Graded Salience Hypothesis. Giora (2008) concludes that metaphor is not unique in that all senses of any word are always activated from the start of processing, and that some activations are faster and stronger than others. This depends on their salience, which has to do with their degree of conventionality and frequency of use, placing them in the forefront of our mind. She notes that, in the case of metaphor-motivated polysemy, many literal meanings (pertaining to the source domain) are less fast and less strong than figurative senses (pertaining to the target domain) for the same word. This suggests that figurative senses are easily available and accessible. They can even be preferred when lexical disambiguation projects the target-oriented situation model from the surface text and text base, and do not require *ad-hoc* construction of a target-domain referent by some form of analogy or cross-domain mapping.

This is not what was originally concluded from the wealth of data collected for testing Conceptual Metaphor Theory. What was concluded there, instead, was that metaphors needed cross-domain mappings for their comprehension (*cf.* Holyoak and Stamenković, 2018). They include data from studies utilizing many different methods and techniques, such as ratings of sensibility of different expressions, formulation of mental images for different expressions, priming data, true-false judgments, comprehension times, and so on.

In the past decade, the interpretation of these data has changed, from pertaining to metaphor *comprehension* to metaphor *understanding* (Gibbs and Colston, 2012: 151; Gibbs, 2017: 85, 212). Thus Gibbs (2017) argues that cross-domain mapping is needed for a full and embodied processing of a metaphor, but he allows for this to take place *after* the more narrow comprehension process has been completed—and the latter clearly may also be based on lexical disambiguation, as is also acknowledged (Gibbs, 2017: 215, 106–111). This suggests that metaphor comprehension based on lexical disambiguation may allow for later (and perhaps optional) metaphor understanding by means of cross-domain mapping, which is precisely how DMT would interpret the data for CMT. This position implies that the various processing data related to lexical disambiguation, categorization, analogy, comparison, and conceptual mapping do not only apply to different structures and functions of metaphors, but that they also may pertain to different moments in the processes of comprehension and understanding (*cf.* Cuccio, 2018). This would resolve the alleged conflict between CMT and DMT completely.

Finally, there are data from other studies that have been interpreted as showing that analogy and cross-domain mapping are active during conventional metaphor comprehension, which according to DMT would as a rule promote lexical disambiguation and categorization instead. These studies may be re-interpreted in a different way (see Steen, 2017). This is not to disparage the value of these studies, because they have obviously revealed important aspects of metaphor processing. However, this is instead to show that they can be given a motivated alternative interpretation within DMT, an alternative which even accords with the above proposals about the difference between comprehension and understanding.

Thus, Steen (2017) argues that some of these studies utilize conventional metaphors that are used deliberately, in for instance studies involving ratings and processing times in Pfaff et al. (1997), experiments on reasoning and decision making in Read et al. (1990) and Robins and Mayer (2000), a study of word and sentence recognition from text in Allbritton et al. (1995), a mental imagery study in Gibbs and Bogdonovich (1999), and an interpretation task in McGlone (1996). Other studies may be re-interpreted as in fact stimulating a deliberate use of conventional metaphors in their task, as in Boers and Littlemore (2000), where participants have to explain conceptual metaphors; Gibbs (1991), which collects verbal explanation of idioms by children; Gibbs (1992), focusing on intuitions about and ratings of metaphorical idioms in five out of six studies; Gibbs et al. (2006), examining people’s reported imaginations of impossible actions; Gibbs et al. (2004), with a questionnaire about people’s knowledge about desire as hunger; Gibbs and O’Brien (1990), examining reported images of idioms; and Nayak and Gibbs (1990), collecting offline appropriateness judgments. In brief, when the task focuses people’s attention on the source domain, this may have triggered deliberate metaphor processing, involving analogy and cross-domain mapping instead of lexical disambiguation, so that the data presented here may in fact count as evidence for DMT.

In conclusion, old processing data can be recruited as evidence for DMT in three ways. First of all, data in favor of the Career of Metaphor Theory can also count as support for DMT, since the Career of Metaphor Theory can be seen as a special case or even one foundation of DMT. Second, data in favor of CMT have been re-interpreted in recent years as possibly pertaining to metaphor understanding rather than comprehension, and this can be seen as complementary to and even compatible with DMT instead of in conflict with it, since understanding is a post-comprehension process. And third, data that have originally been interpreted as pertaining to conventional metaphor comprehension by cross-domain mapping can be seen as the effect of deliberate metaphor use rather than non-deliberate metaphor use because of the materials or tasks used in the experimental research. In all, there is a substantial amount of “old” processing data that can be accounted for in new ways by DMT.

Turning to “old” data on the structures and functions of metaphor that can be related to metaphor and its deliberate or non-deliberate use, it can be easily observed that these are scant. Corpus work on the distribution of metaphor signals and its linguistic forms was virtually non-existent, as was corpus work on novel versus conventional metaphor. This type of research took off when MIP (Pragglejaz Group, 2007) and MIPVU (Steen et al., 2010) were published, and this research emerged at the same time as the first proposal of DMT (Steen, 2008). We therefore now have to turn to new data.

### 4.2. Data since DMT

The influence of deliberate and non-deliberate metaphor use on text comprehension was addressed by a range of studies. Jansen et al. (2010) examined the effects of two alternative deliberate metaphors in a text about HIV/AIDS and the immune system, which would require an army or fire brigade to defend itself; they found that aspects of deliberate metaphor recognition by the participants affected their text understanding, appreciation, and persuasion in comparison with a text version without such a deliberate metaphor. Krennmayr et al. (2014) tested whether deliberate metaphors about economic competition as auto racing (in the form of novel metaphors and signaled metaphors) had a greater effect on memory for various text versions than non-deliberate metaphors (conventional metaphors and non-signaled metaphors); they reported that novel metaphor had a greater influence on recall than conventional metaphor, and that metaphor signaling trended in this direction. Musolff (2016b) studied how students from 10 different countries interpreted the deliberate metaphor of their country as a body or a person and presented the variation in his findings as support for DMT.

A distinct line of research emerged in an exchange with Thibodeau and Boroditsky (2013), who found different framing effects of two diverging *A is B* metaphors in a text on crime (*crime is a beast/virus*) on the preferences expressed by participants for political measures against rising crime in a fictive town, Addison. Steen et al. (2014) critiqued this study on the grounds of its methods, ran an expanded version of it, and could not replicate the effect. As a result, Thibodeau and Boroditsky (2015) designed a new experiment and, again, were able to demonstrate the framing effect of the *A is B* metaphors. Since conventional *A is B* metaphors may be interpreted as deliberate, but not necessarily so since they can also be seen as ambiguous, this may be one reason why not all studies pointed in the same direction.

This in turn led to two further studies, which focused more explicitly on the effects of deliberate metaphor use on political preferences by means of their extension in the rest of the text. One was by Reijnierse et al. (2015), which did not find clear effects, although there was a trend in the right direction. The other was by Thibodeau (2016), which demonstrated a clear effect of extension when the political preferences in the response materials were consistent with those expressed in the text, but which did not have an effect when they were inconsistent. Deliberate metaphor by extension seemed to influence text comprehension when considering its application in executing the task of deciding on political preferences, but did not seem to work equally well in both studies. Again, further research is required to see when, precisely, deliberate metaphors have the predicted effects (see also Hart, 2017, 2020).

Other tasks than the completion of surveys can be found in Thonus and Hewett (2016), De Vries et al. (2018) and Werkmann Horvat et al. (2023), and Silvestre-López et al. (2021). Thonus and Hewett trained half of a group of writing consultants in student writing centers in the deliberate use of metaphor when giving feedback, while leaving the other half of the group of consultants without training; they found that training indeed did have an effect of deliberate metaphor use in the consultants’ production of feedback. De Vries and her colleagues studied eye movements of participants reading two short stories and found that deliberate metaphors were awarded more time than non-deliberate metaphors, which in turn were given less time than non-metaphorical words. Ana Werkmann Horvat and her colleagues utilized eye-tracking in combination with a forced-choice semantic relatedness task to demonstrate that the source domain of conventional metaphorical expressions can be activated when they are supported by further source-domain material in the form of a simile in the rest of the sentence, in contrast with when this does not happen. Silvestre-López and his colleagues designed four conditions in guided meditations that included a contrast between deliberate and non-deliberate metaphor use (versus no metaphors versus no text at all), and found that the deliberate metaphor conditions influenced self-reports of metathinking activity and affective state. Experimental data for deliberate metaphor use are hence growing across a range of methods and techniques including tasks and materials.

The above processing data are a modest but promising basis for further behavioral research. When it comes to structural-functional data, the findings are more encompassing. This is because DMT may also be formulated as a matter of mandatory attention to the source domain as a result of the Grammatical Concordance Hypothesis (Cuccio, 2018). Thus, when metaphors are expressed at the level of discourse, or when they are direct, novel, or signaled, they demand the addressee’s attention to the source domain as part of the referential meaning of the utterance. You cannot comprehend ‘Shall I compare thee to a summer’s day?’ without awarding separate referential attention to the summer’s day as a summer’s day. As a result, DMT research on deliberate metaphor has focused on the distribution and purpose of deliberate metaphor expressed in these particular structures and functions in a wide range of studies (Steen, 2023).

Some of this research is based on the application of a reliable method for general metaphor identification, called MIP (Steen et al., 2010), but initially had to make do with more subjective operationalizations of deliberate metaphor. With the advent of DMIP (Reijnierse et al., 2018), however, the identification and analysis of deliberate and non-deliberate metaphor in the structures and functions of natural language use and discourse has become quite reliable and valid. Its relation to the processes and products of real deliberate and non-deliberate metaphor use remains an empirical issue, as noted before.

A review of the available data can be found in Steen (2023). Corpus work such as Tay (2016) and Reijnierse et al. (2019) has focused on associations between metaphor properties like signaling (language), conventionality (thought), directness (reference), and deliberateness (communication), and found the predicted tendencies back in general patterns of language use. Distributions of these patterns across word classes and registers were also described (Reijnierse et al., 2019), with different distributions for the interaction between metaphor and word class in various registers for deliberate metaphors versus non-deliberate metaphors. Other studies have focused on the connection between deliberate and non-deliberate metaphor, on the one hand, and aspects of discourse domain, on the other. For instance, in politics, several studies demonstrated how deliberate metaphors clearly did other jobs than non-deliberate ones (Perrez and Reuchamps, 2014; Heyvaert, 2019; Heyvaert et al., 2020). Mujagić (2018, 2022) and Mujagić and Berberović (2019) has shown the same point for metaphor in media coverage of the issue of immigration. Other domains that have been described in this way include science (e.g., Navarro i Ferrando, 2016) and education (e.g., Jiménez Muňoz and Lahuerta Martínez, 2017; Cuberos et al., 2019). This is just a selection of the available research.

The comprehension and understanding of these types of metaphor in this type of context needs further experimental work. This is where process theory and research meets with structural-functional theory and research. This is where variation in metaphor structure and function interacts with variation in metaphor processing. This is the heart of DMT.

## 5. Discussion: thinking by metaphor, fast and slow

The basic question asked in DMT is this: do language users intend their metaphor to refer to some source-domain concepts as distinct aspects in the situation they project from their utterance, or not? If they do, then the utterance requires analogical processing in order to integrate the source domain within the situation model, which is about the target domain. Such a source domain typically functions as an alien perspective in a comparison, “alien” because the comparison is deliberately figurative. To DMT, metaphor use is about intended comparison between unlike things.

When a cross-domain comparison is intended as part of the referential meaning of the utterance, we are dealing with deliberate metaphor use. There are plenty of data showing that this requires analogical processing and that this typically happens with signaled, novel, and direct metaphors within utterances (at the level of language use). This is metaphorical thinking. It probably always happens with metaphors that are expressed as metaphors across utterances, at the level of discourse, but experimental research is scant here. At the same time, this type of metaphor use is quite infrequent.

If language users do not intend to refer to some source-domain concepts as distinct entities in the situation they project from their utterance, then their use of metaphor is non-deliberate. They do use a metaphor, but it only emerges via polysemy in the surface text and as corresponding source-domain sub concepts in the text base. This may even trigger short-lived early source-domain activation in the brain, which may be called metaphoric thought. Then lexical disambiguation kicks in, however, and the situation model gets constructed in terms of referents that only pertain to the target domain. There are plenty of data showing that this typically happens with non-signaled, conventional, and indirect metaphors, and it probably never happens with metaphors that are expressed as metaphors at the level of discourse. This type of metaphor use is the rule.

There are many other factors that can clearly play a role in the intentions to use a metaphor as a metaphor. They for instance have to do with characteristics of the participants, who may be sensitive to specific ways of communicating about important topics, such as serious illnesses. They may also concern expectations about the discourse domain within which a metaphor occurs, such as the common idea that literary texts promote metaphorical language use whereas scientific texts do the opposite. These and other factors can be brought together in a theory of genre about discourse events, which is one separate but fundamental issue in coming to terms with the factor of level of cognition in DMT (Steen, 2023), which is all about the interaction between variation in structures and variation in processes during comprehension and understanding.

All in all then, DMT was motivated to solve a problem in metaphor studies that was called the paradox of metaphor, and it looks as if it is on course in successfully addressing this. This is also because DMT was grounded in the general factor of ‘level of cognition’, which assumes a general distinction between language use and discourse that can be applied to reveal distinct aspects of metaphor and its comprehension. It is therefore now time to turn to yet another factor in the framework, that is, speed of cognition. This was introduced to frame DMT as a theory that can break new grounds in metaphor research, and can lead to new applications of it by slowing metaphor down (Steen, 2023).

With the publication of Kahneman’s (2011) *Thinking, fast and slow*, a new way of looking at human cognition was made more possible. He suggests that cognitive scientists should consider thinking from two perspectives, which he dubbed as two systems, one fast and automatic and one slow and controlled with more volition and sometimes awareness (Kahneman, 2011: 21):

I describe System 1 as effortlessly originating impressions and feelings that are the main source of the explicit beliefs and deliberate choices of System 2. The automatic operations of System 1 generate surprisingly complex patterns of ideas, but only the slower System 2 can construct thoughts in an orderly series of steps. I also describe circumstances in which System 2 takes over, overruling the freewheeling impulses and associations of System 1.

Even though there has been criticism of Kahneman’s proposal, his ideas have facilitated new ways of thinking that can also be fruitfully applied to metaphor comprehension (and its production and related processes). They moreover tally well with the independent elaboration of the CI model by Gambi and Pickering (2011), who also speak of an automatic versus an intentional system of cognition.

Metaphor can be comprehended fast and automatically. But it can also be comprehended a little less fast (Steen, 2023), and this raises the question when less fast becomes slow. Kahneman’s theory of problem solving at different speeds and involving different systems of cognition can lead the way here to reconsider how speed of metaphor comprehension is important for theories of metaphor, and in particular for DMT. That metaphor processing can be related to problem solving was pointed out by for instance Kintsch (2008).

DMT holds that thinking by metaphor can be fast and slow (Steen, 2023). It is always fast when it relates to metaphor processing by lexical disambiguation, since that is typically a fully automatic and unconscious process. This would be the case for non-deliberate metaphor processing, which is the rule. It will involve metaphoric thought, in the surface text and text base, but it does not exhibit metaphorical thinking, in the situation model and context model.

Thinking by metaphor can be a little less fast but still automatic and unconscious when people successfully comprehend deliberate metaphors in otherwise neutral contexts. The data from the experiments comparing novel with conventional metaphors, or metaphors with similes, show that this is possible. Generally, analogizing and making comparisons can go fast and without shifting gears in cognition from automatic to less automatic. This is metaphorical thinking, as opposed to metaphoric thought, and it is fast. This is the exception to the rule.

Metaphorical thinking can slow down, however, in several cases. Some novel metaphors may be too difficult or complex to immediately make sense, for instance. Then people have to work harder to come to a satisfactory representation of the utterance, and this may slow them down. It may lead to metaphor awareness, in that people recognize the identity of the little puzzle they have run up against, which in turn may be related to stimulating metaphor recognition, interpretation, and appreciation. This may then even lead to people’s realizing that they are ‘doing metaphor’, which is a form of metaphor consciousness. All of these options affect the content of their mental representation of a metaphor and what people can do with it. This is the exception to the exception to the rule.

Another way in which thinking by metaphor can slow down is when metaphors are not necessarily novel but ask attention for longer times. This will happen in particular when authors design their texts in such a way that a metaphorical mapping is extended across a number of utterances, or even an entire text. The obvious place where this may be expected to happen is literature. However, science communication also makes use of this device, as I have illustrated in my discussion of Susan Greenfield’s *A day in the life of the brain* (Steen, 2023). But even in live interaction, such as therapy sessions or political debates, metaphor may be exploited at longer time intervals to explore the perspective it may generate on an important topic in the session. This is also an exception to the exception to the rule.

One special form of this type of metaphorical thinking that can be slow is people’s resistance to metaphor. For various reasons, specific discourse participants may not like the use of a particular metaphor in a particular way in a specific discourse situation, and protest. Examples where this may or does happen have been collected at an increasing rate in recent research, in the above-mentioned corpus work as well as in dedicated case studies (e.g., Renardel de Lavalette et al., 2019; Finsen et al., 2021; Wackers et al., 2021). This type of research has potential implications for advice on how to communicate and how to design texts and conversations, and would eventually lead DMT into areas of production and interaction.

All of these are moments when metaphor is slowed down, at least for a while, for examining the purpose of its contribution to the discourse event. This probably does not happen very often, which also seems to be suggested by the data collected so far. But it can happen, it does happen, and the big question is when, how, and why it happens. Also, how does this relate to most moments of metaphor processing, where it does not happen? Moreover, the next question is how this can be exploited better, against the background of what we know about how people use metaphor in most cases. Slowing metaphor down would give people more power over their use of metaphor than has long been acknowledged.

Metaphor may have a lot of power over our thinking and actions, but this is not unlimited. Indeed, it seems possible that slowing metaphor down may give us back some of the power over our thinking by metaphor. It is one other fundamental aspect of DMT to show how this can be related to general structures and functions of metaphor as well as to the general mental processes and products of its comprehension and more broadly its use, deliberate or non-deliberate. In this context, the relation between comprehension and understanding and the possibility of post-comprehension use of conceptual metaphors, as well as the ambiguous status of many indirect conventional metaphors between deliberate and non-deliberate use are two exciting novel topics that have been newly generated by this line of inquiry.

## Data availability statement

The original contributions presented in the study are included in the article/supplementary material, further inquiries can be directed to the corresponding author.

## Author contributions

The author confirms being the sole contributor of this work and has approved it for publication.

## Conflict of interest

The author declares that the research was conducted in the absence of any commercial or financial relationships that could be construed as a potential conflict of interest.

## Publisher’s note

All claims expressed in this article are solely those of the authors and do not necessarily represent those of their affiliated organizations, or those of the publisher, the editors and the reviewers. Any product that may be evaluated in this article, or claim that may be made by its manufacturer, is not guaranteed or endorsed by the publisher.
